# Duplicated Heschl’s gyrus associations with phonological decoding

**DOI:** 10.1007/s00429-024-02831-2

**Published:** 2024-07-16

**Authors:** Mark A. Eckert

**Affiliations:** Department of Otolaryngology – Head and Neck Surgery, Medical University of South Carolina, Charleston, SC 29425 USA

**Keywords:** Sulcal/Gyral variability, Heschl’s Gyrus, Phonological decoding, Template-matching

## Abstract

The reason(s) for why a complete duplication of the left hemisphere Heschl’s gyrus (HG) has been observed in people with reading disability are unclear. This study was designed to replicate and advance understanding of the HG and phonological decoding association, as well as test competing hypotheses that this HG duplication association is specifically localized to the HG or could be due to co-occurring atypical development of other brain regions that support reading and language development. Participants were selected on the basis of having a duplicated left hemisphere HG (*N* = 96) or a single HG (*N* = 96) and matched according to age, sex, and research site in this multi-site study. Duplicated and single HG morphology specific templates were created to determine the extent to which HG sizes were related to phonological decoding within each HG morphology group. The duplicated HG group had significantly lower phonological decoding (*F* = 4.48, *p* = 0.04) but not verbal IQ (*F* = 1.39, *p* = 0.41) compared to the single HG group. In addition, larger HG were significantly associated with lower phonological decoding in the duplicated HG group, with effects driven by the size of the lateral HG after controlling for age, sex, research site, and handedness (*p*s < 0.05). Brain regions that exhibited structural covariance with HG did not clearly explain the HG and phonological decoding associations. Together, the results suggest that presence of a duplicated HG indicates some risk for lower phonological decoding ability compared to verbal IQ, but the reason(s) for this association remain unclear.

## Introduction

Heschl’s gyrus (HG) is the site for primary auditory cortex in humans (Rademacher et al. 1993) and is highly variable in morphology (Penhune et al. 1996; Leonard et al. 1998). This qualitatively unique variability in gross anatomy includes a completely duplicated gyrus that is positioned in a parallel anterolateral direction just posterior to the anterior HG. A duplicated HG, which is present in gestation (Pundir et al. 2016), has generated interest because this morphology is more likely to occur in people with better speech sound learning (Golestani et al. 2007), phonetician expertise (Golestani et al. 2011), and auditory processing expertise (Benner et al. 2017; Schneider et al. 2005). However, a duplicated HG has also been observed in people with dyslexia (Leonard et al. 2001). Specifically, a duplicated left hemisphere HG has been associated with poor phonological decoding (Leonard et al. 2001, 2002). The reason(s) for this morphological association with a foundational ability for becoming a proficient reader are unclear.

A potential link between duplicated HG and reading disability was first observed in a small sample of adult participants who had below average phonological decoding ability, a family history of dyslexia, and were described as having a “recovered dyslexic” profile (Leonard et al. 1993). Subsequent studies demonstrated significant associations between duplicated HG and reading disability in adult and pediatric samples (Leonard et al. 2001, 2002). Participants with a specific phonological decoding impairment had a larger duplicated HG surface area compared to control participants. In contrast, children with poor oral language or verbal abilities were not more likely to exhibit a duplicated HG (Leonard et al. 2002). The duplicated HG was described in those studies as an anatomical risk factor because it was one of multiple different anatomical measures that were additive predictors of a specific phonological processing impairment versus oral language impairment (Leonard and Eckert 2008). In addition, duplicated HG have not always been associated with reading disability (Eckert et al. 2003; Green et al. 1999). Thus, it is unclear if duplicated HG have a direct impact on reading development or occur with atypical development of other brain regions that support the development of reading skills.

The posterior duplicated HG has been thought to exhibit a functional duplication or be an extension of the anterior HG (Leonard et al. 1998; Benner et al. 2017). There is some evidence to support this premise based on one study where tonotopic maps were observed on the anterior and posterior duplicated HG (Da Costa et al. 2011). This observation is also consistent with anterior and posterior HG activity during a tone listening task (Benner et al. 2017). However, Brodmann area 41 cytoarchitecture was not observed in the posterior duplication of a small number of human post-mortem cases (Rademacher et al. 1993). Moreover, the anterior duplicated HG exhibited evidence of increased myelination that would be expected for core auditory cortex in comparison to the posterior HG duplication (Sigalovsky et al. 2006), suggesting that the posterior duplication may instead be composed of auditory belt cortex (Benner et al. 2017). The degree to which the duplicated HG associations with reading disability can be attributed to primary auditory core regions within the anterior duplicated HG and/or belt regions of duplicated HG is unclear.

Disrupted cross-modal mapping was also proposed to explain the duplicated HG observation in people with phonological processing difficulties (Leonard et al. 2002). This hypothesis may be supported by evidence of atypical auditory cortex function in people with reading disability during audiovisual tasks (Blau et al. 2009; Ye et al. 2017). Here, duplicated HG are predicted to occur with atypical development in visual cortex and perhaps atypical auditory cortex connectivity with visual cortex. Significant functional connectivity has been observed between primary auditory and visual cortex (Eckert et al. 2008). In addition, fiber projections have been observed between auditory and visual cortex (Beer et al. 2011), including primary auditory cortex and peripheral area 17 in macaque monkeys (Falchier et al. 2002). Thus, it is possible that atypical development of visual cortex contributes to the development of duplicated HG.

Finally, the duplicated HG association with reading disability has also been hypothesized to reflect enlarged cortical maps for oral language functions (e.g., semantic knowledge) that should be relatively preserved in people with a specific deficit in phonological processing compared to people with oral language impairments (Leonard et al. 2002). This hypothesis may be consistent with associations between better speech sound learning and duplicated HG (Golestani et al. 2007). Speech sound learning appears to engage regions of anterior insula and inferior frontal cortex (Golestani and Zatorre 2004; Alotaibi et al. 2023) that exhibit functional connectivity with auditory cortex (Beckmann et al. 2005; Damoiseaux et al. 2006). Thus, it is also possible that exaggerated connectivity between auditory and frontal cortex contributes to the development of duplicated HG and relatively normal oral language ability when there is risk for impaired phonological processing. That is, rather than a marker of disability, a duplicated HG may be a marker of the ability to compensate for impaired phonological processing.

Each of the hypotheses presented above can be examined with a structural covariance approach that examines the degree to which the size of a duplicated HG is associated with the size of other brain regions. One challenge for structural covariance types of analyses (and functional MRI analyses) involving qualitatively unique sulcal/gyral features is difficulty normalizing these features into a common coordinate space. Automated measures of HG structure can be problematic because spatial normalization typically involves normalization to a target space with a distinct single HG (e.g., in the MNI template). A sulcal/gyral specific template approach has been used to study people with different rhinal sulcus patterns (Xie et al. 2017), but there has been limited application to the study of auditory cortex that is highly variable in sulcal/gyral morphology.

The current study was designed with a sulcal/gyral specific template approach to test the hypothesis that a larger posterior duplicated HG relates to poorer phonological decoding but not verbal ability. This study was also designed to examine if structural variation in other brain regions could explain an association between HG size and phonological decoding, including visual cortex and frontal cortex that might support cross-modal and oral language hypotheses for why duplicated HG have been associated with poor phonological decoding.

## Materials and methods

### Participants and HG morphology sampling

This study included 192 participants (age: *x̄* = 15.19, ± 8.03 years; 34% female) whose de-identified data were contributed to the Dyslexia Data Consortium (www.dyslexiadata.org). The data were collected in accordance with the Declaration of Helsinki with Institutional Review Board approval at 14 different research sites and then de-identified prior to data sharing with Institutional Review Board approval from the Medical University of South Carolina to receive de-identified data. The primary inclusion criterion was the presence of a clearly visible duplicated left HG, which resulted in the identification of 96 cases from a larger set of 1189 images that were examined by author M.A.E. who has established expertise characterizing HG morphology (Eckert et al. 2001, 2002; Leonard et al. 2001, 2002). Participants with a sulcus intermedius that splits HG into two distinct gyri (bifid in Hackett et al. 2001; Figure 1 in Leonard et al. 1998) were not included in the current study because these cases have not been clearly related to reading disability (Altarelli et al. 2014). This common-stem HG phenotype presents medially as a single gyrus that appears as two separate gyri more laterally as the sulcus intermedius deepens from medial to lateral planes of section. This sulcus intermedius morphology differs compared to the duplicated HG phenotype where two completely separate HG are present across the superior temporal gyrus. The frequency of HG duplications relative to the larger sample is lower than the 37% occurrence of duplicated HG in 430 participants (Marie et al. 2015), due at least in part to the selection of duplicated and single HG that were easily identifiable exemplars for each HG type. Participants with a single left HG were then selected using the R package MatchIt (v4.5.0) and Mahalanobis distance criterion to be matched with the duplicated HG cases for age (*t*_(1,190)_ = 0.21, *p* = 0.83), reported biological sex (*X*^2^_(1)_ = 0.00, *p* = 1.00), and research site (*X*^2^_(13)_ = 0.00, *p* = 1.00). Descriptive statistics for each HG group are presented in Table 1. Thus, participants were selected based on auditory cortex morphology and individual differences in behavior were then examined between and within HG morphology groups.

### Behavioral measures

Reading ability, verbal ability, and handedness data were available for this study to determine the extent to which HG groups exhibited reading skill differences, including after accounting for verbal ability and handedness. Pseudoword reading (phonological decoding) and real word reading accuracy were assessed with the Word Attack and Word Identification subtests of the Woodcock–Johnson IIIR or Woodcock Reading Mastery Tests (Woodcock 1973; Woodcock 2001). Verbal ability (verbal IQ) was assessed with the Vocabulary Knowledge subtest or Verbal Comprehensions construct of the Wechsler Intelligence Scales for Children and Wechsler Abbreviated Scales of Intelligence (Wechsler 1999, 2004). Participants were also operationally defined as right-handed if they wrote with their right hand or had an Edinburgh handedness inventory (Oldfield 1971) score > 0. All other participants were defined as non-right-handed.

### Image processing

T1-weighted images were collected across the 14 sites with different imaging systems and sequences (GE, Philips, and Siemens 1.5 T and 3.0 T systems; flip angles: 7 ^o^ to 45^o^; echo times: 1.9 to 6.63 ms; and repetition times: 6 to 2530 ms, with the longer repetition times for inversion recover sequences that had inversion times from 131 to 1100 ms; matrices: 256 × 256, 217 × 181, 195 × 200; slices: 124 to 256; slice thickness: 0.80 to 1.30). These native space and skull-stripped T1-weighted images were denoised and rigidly aligned to the 1 mm resolution MNI template using SPM12.

Whole brain templates were created using T1-weighted images from the single HG and the duplicated HG cases with the ANTS SyN diffeomorphic normalization approach (Avants et al. 2009). This normalization procedure was performed using ten warping steps [Normalization parameters steps 1–10: Cross-correlation metric (mm radius = 4.00); SyN (1, 2, 1); Gaussian regularization (2.00); steps (Step 1: 4 × 10 × 10 × 4; Steps 2–10: 10 × 30 × 30 × 10)]. The rigidly aligned images were averaged and this average image served as the initial normalization target space. The average image from each normalization step served as the target for each subsequent normalization step. Here, the goal was to generate whole brain template images that represented the average space of the 96 cases with a duplicated HG and the average space of the 96 cases with single HG. These templates are available at https://www.dyslexiadata.org/HG.

Log Jacobian images were then calculated from the final linear and non-linear warping parameters to place each rigidly aligned image into the single or duplicated HG template space. The Jacobian values represent the extent to which a voxel had to be distorted to fit to the template. Higher Jacobian values are observed when voxels need to be reduced in volume to fit to a template. Thus, larger Jacobian values within HG would be expected to occur for cases with relatively large Heschl’s gyri. The Jacobian images were then smoothed with a Gaussian kernel (full width at half maximum = 8 mm) for statistical analyses, as in our previous work (Eckert et al. 2022).

The average Jacobian determinant was collected for each of the unsmoothed Jacobian images in the single or duplicated HG template space from within regions of interest (ROI) for the single and duplicated HG. These ROI were obtained by tracing the pial-gray matter boundary on each sagittal image section from where the HG first appear in medial plane of section to the most lateral extent where the anterior and posterior HG sulcal boundaries were visible. A total HG size measure was obtained by averaging the Jacobian values across the entire HG using the SPM toolbox MarsBar (Brett et al. 2002), as consistent with previous studies where HG surface area was collected across the medial to lateral extent of the HG (Leonard et al. 2001, 2002). To examine the spatial specificity of effects from these HG measures, HG size was also collected on each medial to lateral section. [20 single HG and anterior duplication HG sections and 16 posterior duplication sections with a common lateral section across the types of HG (i.e., the most medial position of posterior duplication is more lateral than the most medial position of anterior duplication)].

The structural covariance analyses described in the Statistics section included a covariate to control for total gray matter volume. This volume measurement was obtained first using the CAT12 segmentation function with the default settings to obtain a gray matter segmented image for each participant (Gaser et al. 2022). The voxel-wise gray matter probability values in these images were then summed to obtain a total gray matter volume measurement for each participant.

### Statistics

The R Statistics Language [v4.2.1; (R Core Team 2018)] and SPM12 were used for statistical analyses in this study (Ashburner 2009).

Multiple Imputation. This retrospective multi-site study had missing data for the phonological decoding (9%), real word reading (9%), verbal IQ (29%), and right- versus non-right-handedness (9%) measures. Multiple imputation was used to deal with this missingness (Rubin 1996 Eckert, 2016 #17), as is appropriate for the extent of missingness in this study (Vaden et al. Jr 2020; Vaden et al. 2012). For behavioral comparisons between HG groups, the multiple imputation model included variables and participants that would be included in the analysis models, and specifically included research site, age, sex, phonological decoding, real word reading, verbal IQ, handedness, and single or duplicated HG type variables. HG group-specific multiple imputation was also performed for within HG group analyses, including because the single HG group did not have a posterior duplication HG Jacobian determinant measure. That is, the HG size measures were included in the multiple imputation model for each HG group so that they could be included in associations with the behavioral data. Statistical results involving imputed data were pooled across the 10 imputed datasets, as indicated by _*pooled*_ in the Results section. Complete case, or non-imputed data and results are shown in figures to demonstrate that similar effects were observed for analysis of imputed and non-imputed data.

Analyses for Hypothesis Testing. HG group differences in phonological decoding, real word reading, and verbal IQ were examined using t-test comparisons for these groups with participants that were matched by age, sex, and research site matched groups. Paired t-tests were then used to determine if there were differences in behavior within each HG group, thereby providing a test of whether the phonological decoding and verbal IQ profile of the HG groups differed, as in previous studies (Leonard et al. 2001, 2002).

Pearson correlation was used to examine the extent to which the size of the HG measures was related to individual differences in phonological decoding within HG groups. Follow-up multiple regression analyses were then used to determine the medial to lateral extent where the size of the HG was most strongly related to phonological decoding, after controlling for age, sex, handedness, and research site.

Statistical significance was defined using a *p* < 0.05 threshold for duplicated HG and phonological decoding associations because of previous associations reported between these variables (Leonard et al. 2001; Leonard et al. 2002). Multiple comparison correction was not performed for analyses involving post-hoc correlations between the size of the HG on each section and phonological decoding as these analyses were designed to characterize the relative contribution of medial to lateral planes of section that contributed to an overall HG size association with phonological decoding.

Structural covariance analyses were performed to examine the association between the average HG Jacobian determinant values from the HG ROI and the voxel-wise Jacobian values. These analyses identified brain regions that were significantly associated with the size of each HG. The regression models included research site, age, sex, and total gray matter volume covariates of no interest. Family-wise error correction (*p* < 0.05) was used to deal with multiple voxel-wise comparisons and define statistical significance for these structural covariance analyses. These analyses identified brain regions that were significantly correlated with HG size, which were then examined as potential explanations for HG associations with phonological decoding. Specifically, the average Jacobian values within the clusters associated with left HG Jacobian values were used in multiple regression analyses to determine the extent to which they were also related to phonological decoding in the sample. This approach was designed to optimize detection of brain regions that scaled in size with the HG duplication and that could explain the HG duplication association with phonological decoding, while also controlling for multiple comparisons.

## Results

### Behavioral associations with single and duplicated HG types

**Fig. 1:**
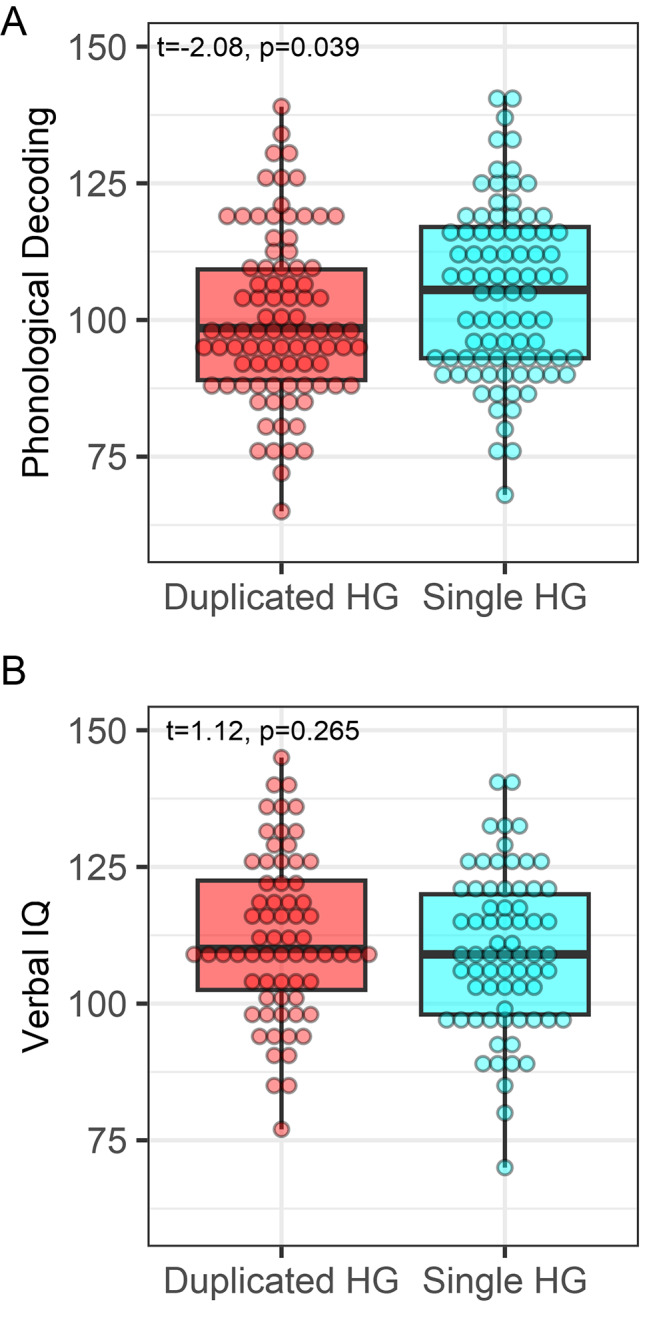
HG morphology group differences. (**A**) Participants with a duplicated HG exhibited lower phonological decoding than those with a single HG. (**B**) No group differences were observed for verbal IQ. Complete case or original non-imputed data and results are shown

**Fig. 2:**
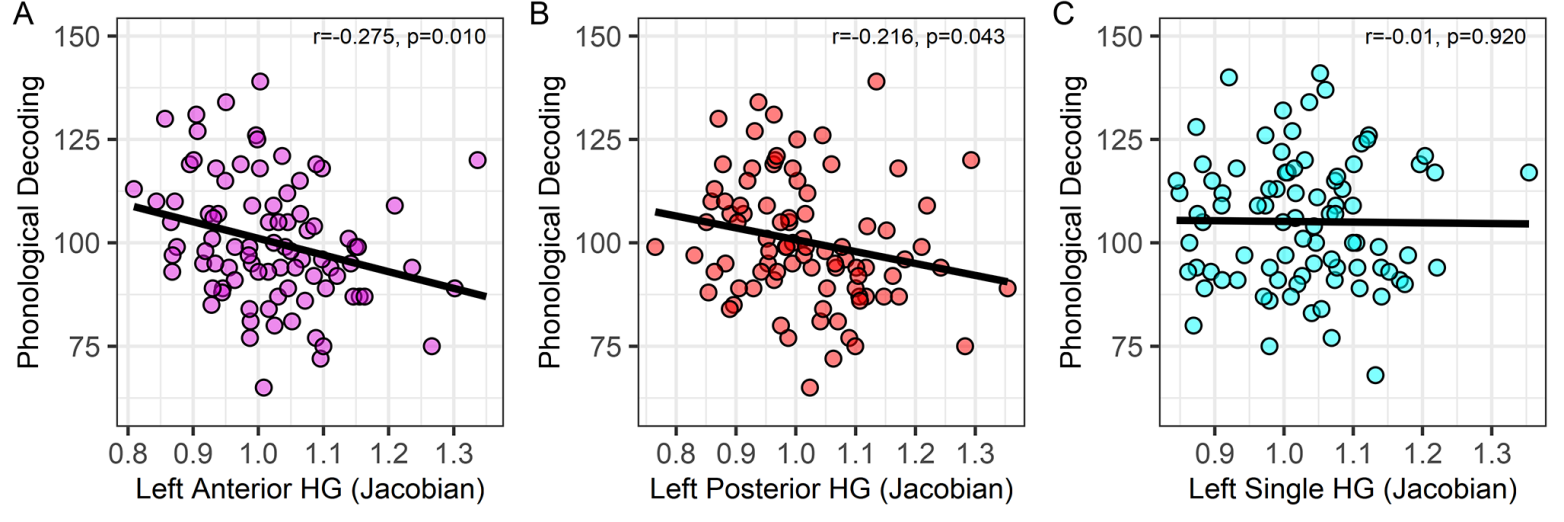
HG size associations with phonological decoding in duplicated and single HG groups. **A**, **B**. Larger anterior and posterior duplicated HG size was associated with lower phonological decoding. **C**. HG size was non-significantly associated with phonological decoding in participants with a single HG. Complete case or original non-imputed data and results are shown

Table 1 shows the distribution of behavioral scores and HG group differences. Table 1; Figs. 1 and 2 also show that participants with a duplicated left HG had significantly lower phonological decoding in comparison to participants with a single left hemisphere HG. Verbal IQ did not significantly differ between the HG groups. These results are consistent with paired t-test results showing that participants with a duplicated HG exhibited significantly lower phonological decoding compared to their verbal IQ (_*pooled*_*t*_(95)_ = -3.85, *p* = 0.0004), whereas participants with a single HG did not exhibit a significant difference in phonological decoding and verbal IQ (_*pooled*_*t*_(95)_ = -1.45, *p* = 0.213).

**Table 1:** HG group behavioral comparisons

	Single HG		Duplicated HG		Group Differences	
	Mean	SD	Mean	SD	*F*	*p* value
Phonological Decoding	104.64	15.50	100.01	15.06	4.48	0.04
Real Word Reading	105.16	18.85	100.43	19.14	3.05	0.10
Verbal IQ	107.51	14.93	109.70	15.29	1.39	0.41

All values are pooled multiply imputed statistics

### HG jacobian size associations with phonological decoding

**Fig. 3:**
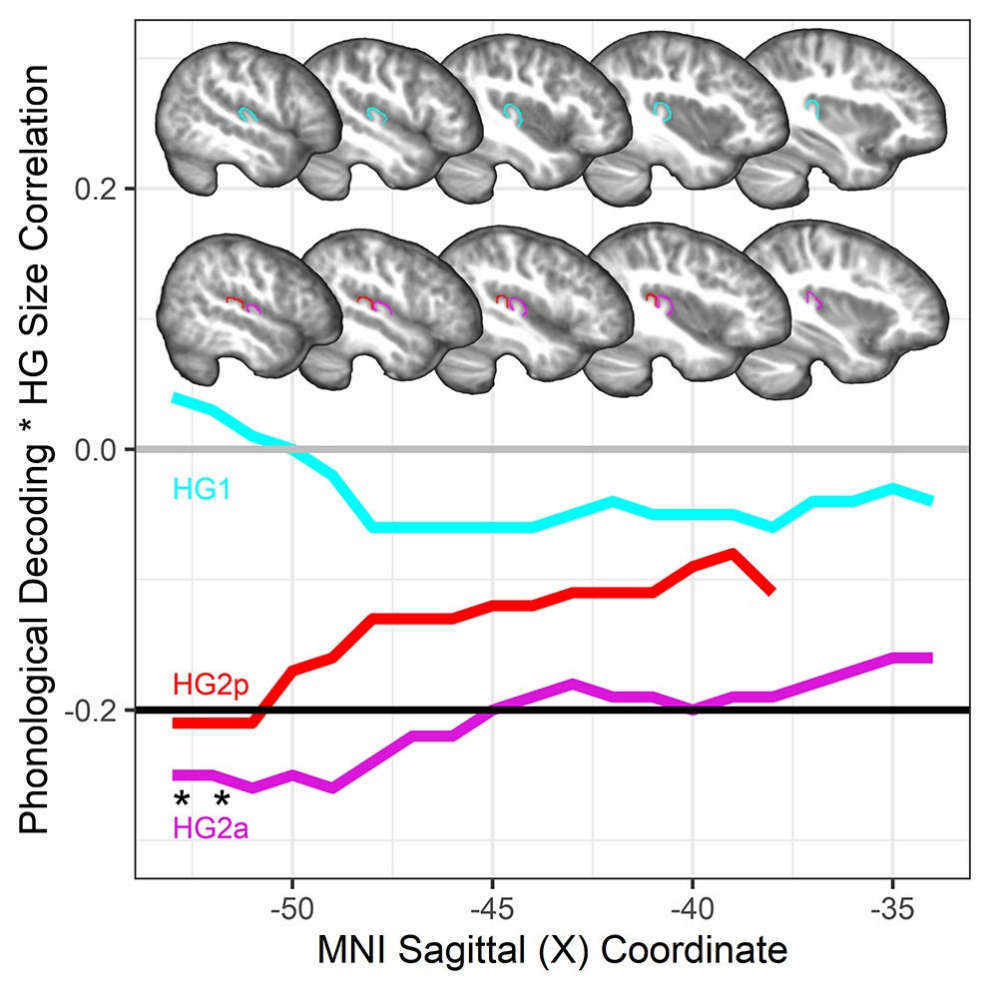
Left hemisphere HG size associations with phonological decoding in single and duplicated HG groups across lateral to medial planes of section (left to right x-axis). Black line indicates r value corresponding to *p* < 0.05. H1: single HG; H2a: anterior duplicated HG; HG2p: posterior duplicated HG. HG2p (red) line does not extend to the most medial X coordinate because H2p appears in more lateral sections relative to H2a and H1. HG group templates and HG ROI corresponding to the MNI coordinates are shown

Table 2 shows the associations between HG size and the behavioral measures within single and duplicated HG groups. Participants with a duplicated HG had lower phonological decoding and real word reading, but not lower verbal IQ, with a larger anterior and posterior HG. Figure 2 shows these negative anterior and posterior duplicated HG size associations with phonological decoding, as well as this non-significant association in the participants with a single HG. Figure 3 shows that the duplicated HG associations with phonological decoding were largely due to HG size in more lateral sections of the temporal lobe compared to medial sections. Multiple regression also demonstrated that the two most lateral sections of the anterior duplicated HG were significantly associated with phonological decoding when controlling for research site, age, sex, and handedness (_*pooled*_*t*s > -2.11, *p*s < 0.05, Cohen’s *d* = 0.48). There were no HG sections that were significantly association with phonological decoding in the single HG group when controlling for research site, age, sex, and handedness (_*pooled*_*t*s < -1.98, *p*s > 0.05, maximum Cohen’s *d* = 0.41).

**Table 2:** Pearson correlation coefficients for the HG jacobian size and behavioral measures

	Phonological Decoding		Real Word Reading		Verbal IQ		Handedness	
	*r*	95% CI	*r*	95% CI	*r*	95% CI	*r*	95% CI
Single HG	-0.01	(-0.21, 0.19)	-0.11	(-0.30, 0.09)	0.06	(-0.15, 0.25)	0.26*	(0.06, 0.44)
Anterior Duplicated HG	-0.29*	(-0.46, -0.09)	-0.33*	(-0.50, -0.14)	-0.07	(-0.27, 0.13)	-0.03	(-0.23, 0.17)
Posterior Duplicated HG	-0.25*	(-0.43, -0.05)	-0.28*	(-0.45, -0.08)	0.01	(-0.19, 0.21)	0.01	(-0.19, 0.21)

* *p* < 0.05, ** *p* < 0.01; All values are pooled multiply imputed statistics. Total gray matter volume did not differ between HG groups (t_(1,190)_ = 1.33, *p* = 0.18) and was not significantly associated with phonological decoding in the duplicated HG group (r_(86)_ = -0.15, *p* = 0.17)

### Specificity of HG association with phonological decoding and structural covariance

**Fig. 4:**
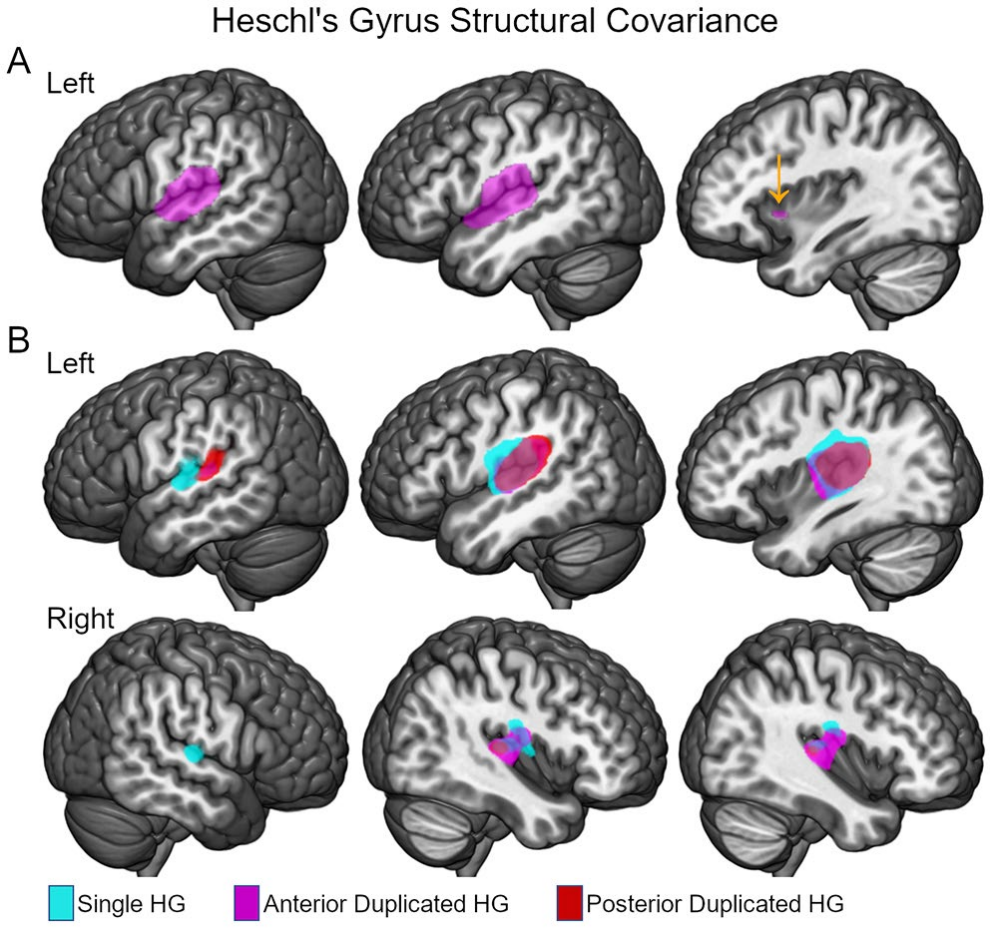
Structural covariance of the left HG. (**A**) The left lateral HG section associated with phonological decoding (Fig. 3: MNI X = -53) increased in size with the size of the left superior temporal gyrus, operculum, and insula / extreme capsule / external capsule. (**B**) Larger size of the entire single, anterior duplicated, and posterior duplicated HG regions of interest were associated with larger right HG. Clusters defined by family-wise error corrected (*p* < 0.05) thresholds for peak voxel effects are shown. MNI coordinates of peak effect correlations with left HG regions of interest [**A**. Left insula/extreme capsule: -37 × 13 x -3; **B**. Right HG: 48 x -11 × 8 (left single HG), 42 x -21 × 9 (left anterior duplicated HG), 46 x -24 × 10 (left posterior duplication)]. These analyses included covariates for research site, age, sex, and total gray matter volume

Structural covariance analysis of the left lateral anterior duplicated HG (MNI X coordinate = -53) demonstrated a relatively confined pattern of covariance around the space of the HG region of interest that extended into the anterior insula, external capsule, claustrum, and extreme capsule. Figure 4 shows that this pattern of structural covariance differed from the structural covariance of the entire HG regions of interest where bilateral HG associations were observed. That is, the more lateral HG region of interest exhibited a more left lateralized and spatially limited association with the size of other brain regions in comparison to the entire HG region of interest. There were no significant negative associations with any of the left HG regions of interest size of other brain regions after correcting for multiple comparisons.

The average Jacobian value within the anterior insula, external capsule, claustrum, and extreme capsule cluster shown in Fig. 4 did not exhibit a significant association with phonological decoding (_*pooled*_*t*_(17,78)_ = -1.92, *p* = 0.07, Cohen’s *d* = 0.44), real word reading (_*pooled*_*t*_(17,78)_ = -0.95, *p* = 0.36, Cohen’s *d* = 0.21), or verbal IQ (_*pooled*_*t*_(17,78)_ = 1.07, *p* = 0.34, Cohen’s *d* = 0.24) after controlling for research site, age, sex, and handedness. A comparison of beta values from these regression analyses also demonstrated that the phonological decoding association with the average Jacobian value in the anterior insula, external capsule, claustrum, and extreme capsule cluster was not significantly different from the association with the anterior duplicated HG association (*Z* = 0.85, *p* = 0.40). In addition, the right HG regions associated with the left anterior and posterior duplicated HG also did not exhibit a significant association with phonological decoding after controlling for research site, age, sex, and handedness (_*pooled*_*t*s ≥ -1.07, *p*s ≥ 0.30). Thus, the competing hypothesis that a duplicated HG association with phonological decoding was due to covariance with another brain region was not clearly supported.

## Discussion

The results of this study replicate previous findings showing a duplicated HG association with lower phonological decoding and not verbal ability (Leonard et al. 2001; Leonard et al. 2002). The current study expands on those findings by showing that the size of the anterior and posterior duplicated HG increased with decreasing phonological decoding across participants with a duplicated HG. These associations were most evident in more lateral sections of the temporal lobe rather than in core primary auditory cortex that is present in more medial regions of HG. The structural covariance analyses also demonstrated that the effects appeared to be relatively specific to the duplicated HG, and perhaps surrounding regions, but did not provide clear evidence that the size of another brain region could explain the duplicated HG association with phonological decoding. These significant results included small to medium effect sizes and thus it is not clear that early developmental expression of a duplicated HG is a causal predictor for relatively specific difficulties learning about letter-sound correspondence.

### Replication of previous HG duplication and reading disability associations

The results of the current study are consistent with previous evidence that people with a relatively specific phonological processing impairment were more likely to have a duplicated HG compared to normative readers (Leonard et al. 2001) and compared to those with specific language impairment (Leonard et al. 2002). The Cohen’s *d* effect sizes in those studies were 0.93 and 0.70, respectively. The Cohen’s *d* effect sizes in the current study for the (1) HG group differences in phonological decoding and (2) the HG size association with phonological decoding within the duplicated HG group were 0.30 and 0.48, respectively. This study differs from previous studies in the selection of participants based on HG morphology rather than based on behavioral inclusion criteria for reading disability. Thus, there may be increased sensitivity to observing HG duplication effects when participants are selected for a relatively specific deficit in phonological processing. However, the effect sizes observed in the current study may be closer to the true effect size given the relatively large sample size compared to those previous studies and because there have been previous studies where a duplicated HG was not significantly associated with reading disability (Eckert et al. 2003; Green et al. 1999). That is, the presence of a duplicated HG appears to be a modest predictor of lower phonological decoding ability, particularly in people with relatively better verbal abilities. Thus, a large sample size appears necessary to observe statistically significant HG morphology and phonological decoding associations.

### Location of effect and potential functional significance

A novel finding from this study was the HG location of the HG size association with phonological decoding. The HG duplication association with phonological decoding in participants with a duplicated HG occurred in more lateral planes of section rather than medial auditory cortex. These results suggest that disruption and/or duplication of auditory field maps in core auditory cortex is not a likely explanation for why a duplicated HG is related to lower phonological decoding. The lateral HG region (e.g., MNI − 53, -14, 4) where there were significant phonological decoding associations has also been associated with elevated activity across a wide range of auditory tasks, including phonological processing tasks (Church et al. 2008; Booth et al. 2008) that have working memory demands (McGettigan et al. 2011) and audiovisual cross-modal tasks (Van Atteveldt et al. 2004; Hertrich et al. 2011). There is also evidence for atypical audiovisual activity in the lateral HG of people with reading disability (Blau et al. 2009). These observations may be consistent with the hypothesis that duplicated HG reflect disrupted cross-modal mapping between auditory and visual cortex (Leonard et al. 2002). However, there were no significant structural covariance results suggesting that the size of the duplicated HG was related to the size of visual cortex regions that might support a cross-modal patterning hypothesis.

The results of this study should also be considered in the context of research demonstrating that people with better speech sound learning (Golestani et al. 2007) and auditory processing expertise [i.e., musicians; (Benner et al. 2017; Schneider et al. 2005)] are more likely to have a duplicated HG. While there are musicians with dyslexia who have low phonological processing abilities (Bishop-Liebler et al. 2014), an anatomical marker of reading disability would not be expected in people with exceptional auditory processing abilities. In one of those studies of musicians, participants with duplicated HG were more likely to have a larger response in the lateral HG to tonal stimuli (Benner et al. 2017) compared to participants with a single HG, including as measured by an early electrophysiology response (P2) in auditory cortex. This response has also been linked to auditory learning [ (Tremblay et al. 2001; Tong et al. 2009); although see (Sheehan et al. 2005) where sound exposure may be sufficient to increase the P2] and was observed to be larger in normative readers compared to reading disability cases (Hämäläinen et al. 2007).

A key to unlocking the reasons for the seemingly different auditory training and expertise results with results of this study may be the oral language abilities of the participants. Again, participants with duplicated HG had significantly higher verbal IQ than their phonological decoding ability in this study. This was not the case in the participants with a single HG. Thus, it is not clear that the duplicated HG is a marker of disability as much as it is a marker of the ability to develop normal verbal ability when there are problems learning letter-sound correspondence. People with duplicated HG may have acute auditory perception and working memory for speech sounds that provide a relative preservation of verbal abilities (i.e., the ability to learn aurally when audiovisual integration is limited). There was significant structural covariance between the lateral anterior HG and the anterior insula / extreme capsule in the duplicated HG group. This region includes projections between temporal and inferior frontal cortex in the macaque (Berke 1960) and has been linked to semantic and phonological functions in humans (Dick and Tremblay 2012). However, there was not strong support for an anterior insula / extreme capsule explanation for the duplicated HG association with phonological decoding, or relatively better verbal IQ in the duplicated HG group.

### HG structural covariance

When limiting the structural covariance results to correlations that survived family-wise error correction for multiple comparisons, there was sparse evidence that size of the duplicated HG was associated with cortical morphology outside of auditory cortex, again, with the exception of anterior insula / extreme capsule, and homologous right hemisphere HG regions when using family-wise error correction for multiple comparisons. The spatial pattern of structural covariance results depended on the HG region of interest. An anterior insula / extreme capsule association was only observed when the lateral HG section association with phonological decoding was examined and not for the entire HG region of interest. In contrast, the average size of the entire HG region of interest was significantly associated with size of the right HG. While interesting that the size of the left hemisphere HG scales with the size of the right HG, and consistent with the functional imaging literature demonstrating functional covariance between homologous regions between hemispheres (Andoh et al. 2015), this right HG observation also did not explain why a left hemisphere duplication was associated with phonological decoding.

### Limitations

This study of retrospective data was limited in the availability of supporting behavioral data across the cases. While multiple imputation was used to deal with missingness, there was inconsistency in data collection across sites. For example, writing hand and quantitative hand preference data were integrated to define and control for potential handedness effects (Marie et al. 2015). Moreover, there was no information available regarding musical experience or auditory processing abilities that might have informed the results.

Research site dummy variables were included in the statistical analyses to address site differences in image acquisition across sites. This conservative approach may have limited HG and phonological decoding association effect sizes to the extent that research sites used different participant sampling approaches, which could have resulted in the inclusion of more participants with a specific deficit in phonological processing at some sites compared to others. One reason for use of the pair matching method in this study was to address this multi-site limitation when comparing single and duplicated HG groups.

The structural covariance results were used to guide statistical tests of the hypothesis that a duplicated HG occurs with atypical development in other brain regions. This approach was taken to optimize the identification of brain regions that exhibited shared variance with HG size and phonological decoding. However, these analyses were not strongly supportive of the hypothesis that atypical development in other brain regions explain the duplicated HG and lower phonological decoding association. Fiber tract and brain activity metrics were not available and could have been useful for testing this hypothesis. For example diffusion metrics of HG and frontal connectivity through the extreme capsule or activity metrics of left inferior frontal cortex support during challenging language tasks (Vaden et al. 2013, 2022) may have be more sensitive and informative than the structural covariance approach.

## Conclusions

People with a duplicated left hemisphere HG were more likely to have lower phonological decoding compared to those with a single left hemisphere HG. This effect was driven by the size of the duplicated HG in lateral planes of section where auditory cortex is responsive to speech stimuli and integrates audiovisual information. However, the HG duplication effect sizes in this study were relatively small. Additional research is necessary to determine if the HG duplication is directly related to problems learning letter-sound correspondence or reflects the potential for normal oral language development despite other developmental risk(s) that contribute to atypical phonological processing.

## Data Availability

The image processing methods used in this study are relatively standard and have been published previously, including with access to the ANTS code used for normalization (Eckert et al. 2019). The data used in this study are available with completion of an established data use agreement, as required by institutional data sharing procedures. Requests can be made directly to the corresponding author or through https://www.dyslexiadata.org. The single and duplicated HG templates are available at https://www.dyslexiadata.org/HG.
